# Pavlovian conditioning and cross-sensitization studies raise challenges to the hypothesis that overeating is an addictive behavior

**DOI:** 10.1038/tp.2014.28

**Published:** 2014-04-29

**Authors:** M R Harb, O F X Almeida

**Affiliations:** 1 NeuroAdaptations Group, Max Planck Institute of Psychiatry, Munich, Germany; 2 Institute of Life & Health Sciences (ICVS), University of Minho, Braga, Portugal

## Abstract

Elevated glucocorticoid levels and sign tracking (ST) in Pavlovian conditioning are potential biomarkers of compulsive behaviors such as addiction. As overeating is sometimes viewed as a form of addictive behavior, we hypothesized that murine Pavlovian sign trackers would have a greater propensity to overeat and develop obesity. Using a food reward in the classical conditioning paradigm, we show that ST behavior is a robust conditioned response but not a predictor of eating and growth trajectories in mice, thus challenging the view that the development of obesity and drug addiction depend on identical mechanisms. This interpretation was supported by experiments which showed that overweight mice do not display cross-sensitization to an addictive drug (morphine), and conversely, that overweight morphine-sensitized animals do not overconsume a highly rewarding food. Although the rewarding/motivational effects of both food and drugs of abuse are mediated by similar neurochemical mechanisms, obesity and drug addiction represent a summation of other dysfunctional input and output pathways that lead to the emergence of two distinct disorders, each of which would deserve a specific pharmacotherapeutic approach.

## Introduction

Overweight and obesity may be direct or indirect antecedents of neuropsychiatric disorders such as depression, anxiety, stroke and dementia;^1,2^ on the other hand, psychiatric conditions and/or certain psychotropic medications may lead to overweight.^3^ Associative learning has an important role in eating behavior and is particularly important in the context of the current obesity epidemic because of the impact of peer pressure and advertising on the formation of eating habits.^4^ Pavlovian (classical) conditioning is a simple form of appetitive learning where individuals develop conditioned responses (CRs) to external cues; over time, the brain implicitly attributes high motivational valence to stimuli that previously carried no value. Although an evolutionarily conserved form of learning that facilitates adaptation, conditioning may underpin maladaptive behaviors, including overeating and obesity.^5,6^ Learned cues can result in overeating by overriding satiety signals^7, 8, 9, 10^ through the activation of brain circuits involved in sensory processing and reward anticipation as well as emotional centers involved in memory and habit formation.^11^

There are three types of CRs: sign tracking (ST), goal tracking (GT) and intermediate tracking (IT, alternate between the location of reward delivery (unconditioned stimulus (US)) and the conditioned stimulus (CS+)). In contrast to GT, ST subjects make more approaches to the CS+ versus US; their ‘cue reactive' behavior is thought to result from attribution of ‘incentive salience to reward cues, transforming predictive conditional stimuli to incentive stimuli with powerful motivational properties'.^12^ Research in animals suggests that ST reflects impairments in behavioral inhibition and vulnerability to drugs and substances of abuse.^13, 14, 15^

Overeating in excess of physiological need has been likened to other compulsive (addictive) behaviors, in particular because anticipation and consumption of food increases dopamine release and dopamine receptor activation in the corticolimbic brain,^16,17^ resembling the neurochemical profile of subjects showing a preference for drugs of abuse.^18^ This overlapping has triggered a lively debate about whether overeating and obesity represent an addictive behavior;^19, 20, 21, 22, 23, 24, 25, 26^ a core tenet of the ‘food addiction hypothesis' is based on the fact that dopaminergic circuits, involved in motivation and reward, are activated in obese and drug-addicted states.^21^ Other support for the ‘addiction hypothesis of obesity' derived from studies in which binging on sugar by rats was interpreted as ‘sugar addiction'.^19,25^ On the other hand, the weaknesses of the experimental paradigms used and the limitations of extrapolating the findings in rodents to humans have been discussed in an instructive review.^22^ The present study approached the question from a different perspective, using the Pavlovian conditioning paradigm. Our results show that ST behavior is not associated with greater consumption of highly rewarding foods and that there is no cross-sensitization between food and the highly addictive psychoactive drug (morphine).

## Materials and Methods

### Animals

Experiments conformed to local and national ethical guidelines, including the precepts of European Union Directive 2010/63/EU. Male C57BL6 mice (Charles River, Sulzfeld, Germany), aged 3–4 months, were used; mice were housed in pairs under standard laboratory conditions. All diets were from Charles River. Behavioral tests were conducted during the animals' activity phase after 1 week of habituation (room, experimenter, calorie-restriction schedule to induce 10–15% loss of original body weight); unless otherwise stated, the calorie-restriction schedule was applied throughout; mice had *ad libitum* access to water. In some experiments, variable degrees of overweight were induced by maintaining mice on either a standard laboratory (normal) chow, low-fat diet (LFD, # D12450B; 16.1 J g^−1^, 10% from fat, 70% from carbohydrate) or a high-fat diet (HFD, D12451; 19.8 J g^−1^, 45% from fat, 35% from carbohydrate). Diet-induced obesity was induced over 3 months by maintaining animals on HFD.

### Pavlovian conditioning

Autoshaping was performed in automated touchscreen chambers, as previously described.^27^ The conditioned stimulus (CS) was a 10 s flash of white light in either the left- (50% of animals) or right- (50% of animals) hand side of the screen. Immediately after stimulus offset, a liquid food reward (15 μl of diluted condensed milk (14% sugar), low-fat cream (5% fat, 8.7 kJ g^−1^) or high-fat cream (32% fat, 13 kJ g^−1^)) was delivered into the food magazine.

During task acquisition mice were trained to associate the light stimulus (CS+) with reward delivery. During each session (one per day), presentations of 15 CS+ and 15 CS− were made in a randomized order (maximum of two consecutive presentations of same stimulus, VI schedule of 10–40 s between each stimulus). Animals reaching criterion (70% of correct (CS+) approach responses/session on at least 3 consecutive days) were designated as ST. Retention was tested 2 weeks after the acquisition phase (all test conditions as during acquisition phase) in satiated animals (3 consecutive days of food *ad libitum*). Extinction of CRs commenced 1 day after completion of retention testing. All test parameters were as before except that approaches to the CS+ were not rewarded, and training sessions were conducted until mice made an equal number of CS+ (15) and CS− (15) approaches over at least two consecutive sessions. In each session, (i) number of CS+ and CS− approaches, (ii) mean latency to approach CS+ and CS−, (iii) mean latency to collect food reward following correct responses and (iv) session completion time, were recorded.

### Test of motivational state

This test (independent of learning strategies) was carried out over 2 days in the touchscreen chambers (reward: 15 μl of milk containing 14% sugar). The latency to retrieve all of the reward and number of food-tray entries were monitored in each session (15 reward cycles, delivered with a VI of 10–40 s).

### Tests of emotionality

All the tests were performed during the daily phase of activity (lights off in animal housing room). The open field (OF) test was used to measure locomotor activity and explorative behavior, 4 weeks after autoshaping; animals were housed as before, with food and water available *ad libitum*. The OF arena was made of Plexiglass (white base: 30 × 30 cm; dark gray walls: 30 cm high). Testing was done in a dark room but the arena was uniformly illuminated with white light (100 lux). Activity of the mice was recorded using a video camera and the results were subsequently analyzed using ANY-maze software (Stoelting, Wood Dale, IL, USA). Mice were tested in the apparatus for 5 min, and the total distance traveled and time spent in the center was computed for each mouse.

Mice were subsequently habituated to the OF apparatus over 2 more days before being subjected to a novel object test to examine reactivity to novelty. The novel object was a small plastic toy placed in the center of the OF; video-tracking with ANY-maze software was used to measure interaction times with this unfamiliar object.

Stress-coping behavior was analyzed in a one-session version of the forced-swim test (FST) by monitoring floating versus swimming time (higher floating time indicated better stress-coping strategy). The FST apparatus consisted of an acrylic glass cylinder (dimensions: height × radius: 60 × 15 cm) filled with tap water (25 °C); the water was changed between every trial. Animals were placed in the cylinder for a total of 6 min, but behavior was recorded during the last 4 min only. Testing was carried out in a dark room, but the FST cylinder was directly illuminated with white light (80–100 lux). Floating and swimming times were recorded by video camera and videos were analyzed with ANY-maze software. Mice that were immobile, with movements of only the hind legs to maintain balance, were considered to be floating; use of tail movements to maintain the head above water was scored as swimming behavior.

### Neuroendocrine response to stress

The dynamic reaction of the hypothalamo-pituitary-adrenal axis to an acute stress (vortexing in 200 ml glass beaker, 2 min) was evaluated by measuring blood corticosterone (125I-Corticosterone RIA kit, ICN Biochemicals, Costa Mesa, CA, USA) in ventral tail vein samples collected at intervals for up to 120 min.

### Morphine cross-sensitization in mice of differing body masses

Mice maintained on either a standard laboratory diet, LFD or HFD were subjected to a slightly modified version of a previously published cross-sensitization protocol,^28^ schematized in Figure 5a.

### Sucrose consumption test

Mice that had been satiated on either LFD or HFD were given a choice between water and a 5% sucrose drinking solutions. Fluid consumption of the mice was measured at 3, 6 and 24 h thereafter; importantly, food was available *ad libitum* throughout, allowing assessment of the hedonic value of the highly-rewarding sucrose solution, independently of the animal's state of satiety or energetic needs. Immediately thereafter, animals were food-deprived for 24 h and provided the water-sucrose choice, allowing discrimination between sucrose consumption due to hedonic liking versus energetic needs.

### Data analysis

Statistical analysis was performed with Prism 5.0 statistical package (GraphPad, La Jolla, CA, USA). Data were first subjected to one- or two-way analysis of variance, followed by Bonferroni-corrected post-test comparisons. The level of significance was set at *P*<0.05.

## Results

### Divergent CRs do not imply differences in learning ability

Associative learning has an important role in the shaping of eating and other behaviors. After conditioning, the brain implicitly attributes high motivational valence to a previously neutral stimulus. Here, calorie-restricted male mice were rewarded with a liquid food (sweetened milk) in the Pavlovian conditioning paradigm in which light served as the neutral stimulus. On the basis of their CR on the last 3 days of conditioning, mice were categorized as STs (minimum 65% approaches to CS+), GT (less than 20% approaches to CS+), or IT (20–65% approaches to CS+).

**Figure 1:**
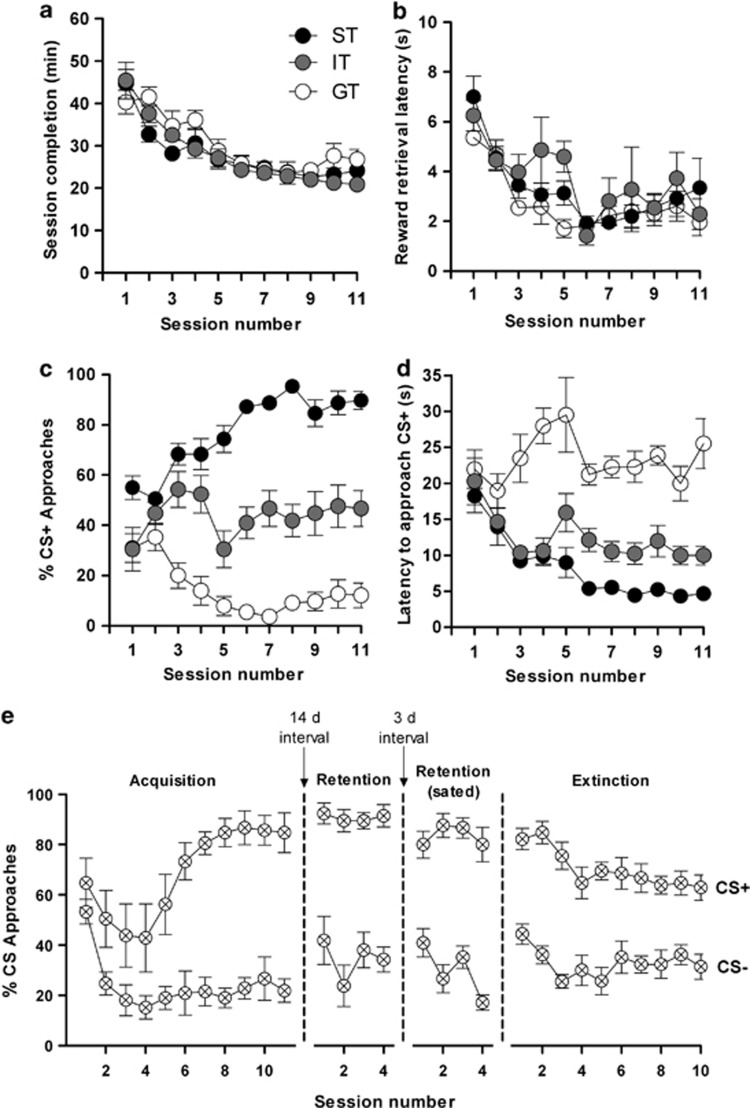
Acquisition of conditioned responses. Mice displayed different conditioned responses, sign-tracking (ST, predominantly approached the CS+ *n*=13), goal-tracking (GT, predominantly approached the US; *n*=11) and intermediate-tracking (IT, alternated between CS+ and US with approximately equal frequency; *n*=7). Autoshaping was monitored over 11 sessions; in each session, mice received 15 CS+ and 15 CS− presentations. (**a)** Time (min) for completion of session. (**b**) Mean latency (s) to retrieve the food reward. (**c**) Relative number of CS+ approaches during each session. (**d**) Mean latency (s) to approach the CS+ during each session. (**e**) Results derive from a cohort of ST mice (*n*=7) different to that used in upper panels (**a**–**d**). Left-most panel shows acquisition of the task (11 sessions). After a 14-day interval, mice were tested for retention of their conditioned responses under standard testing conditions involving food restriction (second-from-left panel) and, following a 3-day interval, retention was tested when mice were fed *ad libitum* (second-from-left panel). The left-most panel depicts results of an experiment to test extinction of the conditioned response; over 10 consecutive sessions. Data shown are means±s.e.m. CS, conditioned stimulus; US, unconditioned stimulus.

None of the mice differed in learning ability, as judged by time to complete the sessions (Figure 1a) or reward retrieval latency (Figure 1b). Notably, all animals required progressively shorter times to complete the task (F_10,303_=26.5; *P*⩽0.0001) (Figure 1a). Absence of learning impairments was further confirmed by data on the relative number of approaches (Supplementary Figure 1A) and total number of approaches (Supplementary Figure 1B) towards the CS−, as well as the latency to approach the CS− (Supplementary Figure 1C). Interestingly, 42, 35 and 23% of the mice showed segregation into ST, GT and IT behaviors, respectively, (F_2,303_=409.8; *P*<0.0001) (Figure 1c). The ST and GT animals consistently showed significant differences between sessions 3 and 11 (*P*⩽0.001) and, although the ST group made progressively more CS+ approaches, the number of CS+ approaches by GT animals steadily declined over successive test sessions. In contrast to ST and GT mice, IT mice alternated between the CS+ and CS− with similar frequencies during sessions 3–11 (*P*⩽0.001 versus ST and GT groups) (Figure 1c). Overall, significant differences were also seen in the total number of CS+ approaches (F_10,303_=4.6; *P*⩽0.0001) and all groups differed from one another in terms of overall CS+ approaches ([F_2,303_=51.2; *P*⩽0.0001) (Supplementary Figure 1D). Lastly, ST, GT and IT differed significantly in their latencies to approach the CS+ (F_2,300_=138.61; *P*⩽0.0001) (Figure 1d); notably, compared with ST animals, GT animals showed higher latencies to approach CS+ (*P*<0.001) (Figure 1d).

Strength of conditioning was demonstrated by testing retention in a separate set of ST animals. As shown in Figure 1e, the relative number of CS+ and CS− approaches on the first day of testing (session 1 in second-from-left panel of Figure 1e) did not differ significantly from that observed in the last session (session 11 in left-hand panel of Figure 1e); importantly, however, ST mice approached the CS+ more than CS− (F_1,48_=167.1; *P*⩽0.0001). Further, the same retention patterns were observed when, animals were tested for their CS approaches when satiated (that is, 3 days food *ad libitum*); as shown in Figure 1e (third panel from left), the relative number of CS+ versus CS− approaches were significantly different (F_1,48_=223.1; *P*⩽0.0001). Next, we asked if the conditioned memory could be extinguished by reward nondelivery. As depicted in Figure 1e (right-hand panel), ST mice continued to make more CS+ than CS− approaches (F_1,120_=276.8*; P*⩽0.0001) during all 10 sessions.

Briefly, this set of experiments demonstrates that mice adopt ST, GT or IT behaviors (CR) which, nevertheless, do not reflect differences in learning ability. Moreover, the CR of ST mice to a food reward are robustly retained, hard to extinguish and persist even when the animals are food-satiated.

### Segregated learning curves persist independently of reward value

Learning is strongly influenced by motivational state.^29,30^ The latter increases as the subjective or real value of a reward increases and sweet and fatty foods carry particularly high incentive salience for many species, including mice.^31^

**Figure 2:**
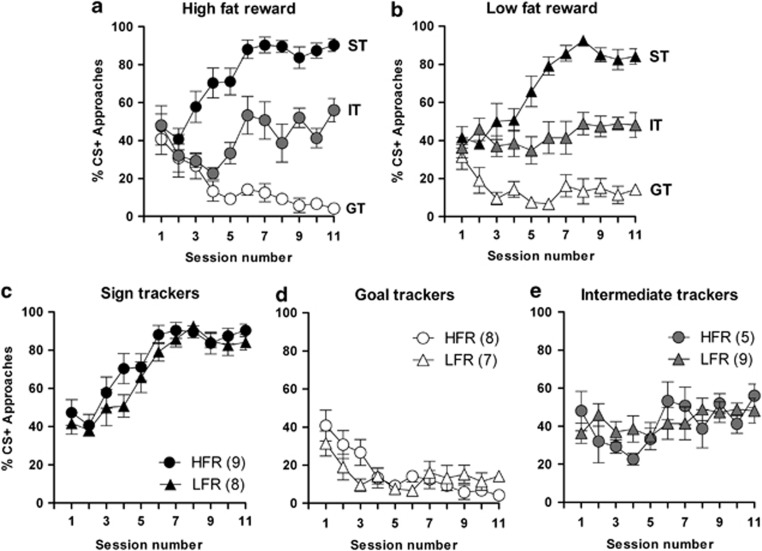
Conditioned responses do not shift with changes in reward value. Shown are the approaches to the CS+ in each of the 11 test sessions consisting of 15 CS+ and 15 CS− presentations. (**a**) Mice rewarded with a high-fat reward segregated into sign trackers (ST; *n*=9), goal-trackers (GT; *n*=8) and intermediate trackers (IT; *n*=5). (**b**) Mice rewarded with a low-fat liquid reward segregated into sign trackers (ST; *n*=8), goal-trackers (GT; *n*=7) and intermediate trackers (IT; *n*=9). Acquisition of ST, GT and IT conditioned responses to high-fat or low-fat rewards is shown in (**c**, **d** and **e)**, respectively. Data are presented as means±s.e.m. CS, conditioned stimulus.

Mice were assigned to receive either a low-fat (5%, *n*=24) or high-fat (32%, *n*=22) reward during Pavlovian conditioning. By clustering animals according to their acquisition of the task we observed segregation of animals into ST, GT and IT groups, irrespective of the conditioning reward (Figure 2a and b) (high-fat reward: F_2,209_=248.9; *P*⩽0.0001; low-fat reward: F_2,231_=238.9; *P*⩽0.0001). Reward value (high-fat versus low-fat) did not alter the rate of CR acquisition by ST (Figure 2c) and GT (Figure 2d) mice, with ST and GT animals respectively displaying gradual increases (F_10,165_=22.4; *P*⩽0.0001) and decreases (F_10,143_=6.02; *P*⩽0.0001) in approaches to the CS+ over time. As expected, IT mice fluctuated between CS+ and CS−, irrespective of the value of the reward (Figure 2e). Thus, individual expressions of ST, GT and IT CRs evolve independently of reward value.

### ST does not predict impaired emotionality or responses to stress

Associations between ST behavior, susceptibility to addictive behaviors and exaggerated corticosterone responses to stress and hyperemotionality were reported previously.^32^ST, GT and IT CR are commonly observed in Pavlovian conditioning^33,34^ and ST, in association with hyperemotionality and exaggerated responses to stress, is suggested to presage addictive behavior.^6^ We here asked, Is ST behavior *per se* a general predictor of dysregulated behavior?

**Figure 3:**
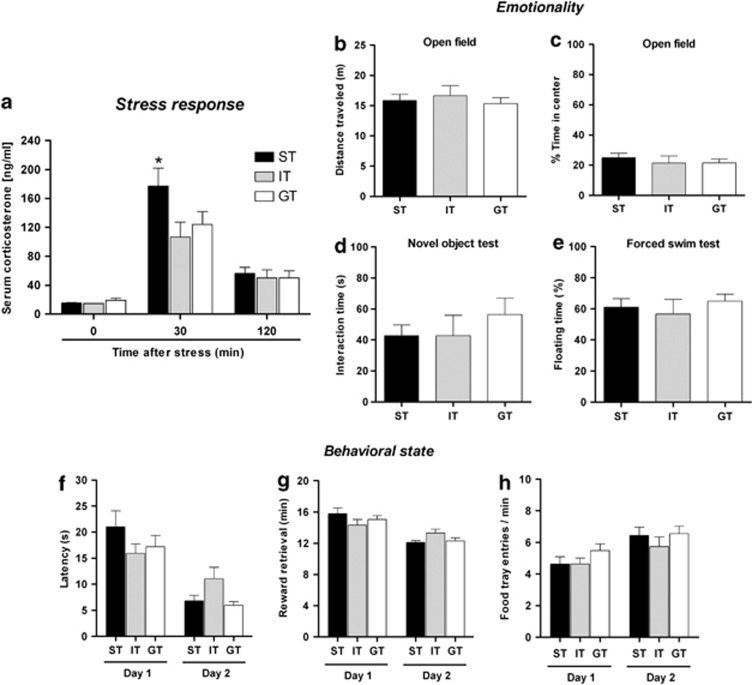
(**a**–**d**) Stress-coping and emotional phenotype in sign-tracking (ST), goal-tracking (GT) and intermediate-tracking (IT) mice. Tests were performed in ST (*n*=13), GT (*n*=11) and IT (*n*=7) mice. (**a**) Serum corticosterone levels in mice under basal conditions (0 min after stress) and 30 and 120 min following an acute stressor (see Materials and Methods for experimental details) are depicted. Note that although ST mice showed the most robust hormonal response to the stressor, all animals had similar levels of corticosterone 120 min post stress, indicating that glucocorticoid negative feedback mechanisms were unimpaired in the ST group. (**b**–**c**) Locomotor activity was monitored in an open field arena. Two parameters were monitored: total distance traveled (m) and time spent in the center of the arena during a 5-min test period. (**d**) The novel object test was used to assess emotionality in terms of time spent exploring an unfamiliar object placed in the center of an open field arena. Interactions of ST, GT and IT mice with the novel object were monitored over 5 min. (**e**) Stress-coping behavior in ST, GT and IT mice was compared in a one-session forced-swim test. All groups of mice showed similar times spent floating, that is, showed identical stress-coping capacities. The depicted data are means±s.e.m; the asterisk indicates a higher value in ST (*P*<0.05), compared with GT and IT. (**f**–**h**) Motivation for food reward does not differ between ST, GT and IT mice. Animals were rewarded with sweetened milk, considered to be more rewarding than their standard food pellets. Shown are the mean latency to approach the reward (**f**), the time taken to retrieve (and consume) the food reward (**g**), and the number of food-tray entries (**h**) by ST, GT and IT mice. Measurements were made over two sessions, with 15 reward deliveries in each. The results are shown as means±s.e.m.

Previously-designated ST, GT and IT mice (Figure 1) showed similar baseline levels of corticosterone (Figure 3a) and responded to a brief stressor with increased corticosterone secretion within 30 min (F_2,81_=53.7; *P*⩽0.0001) (Figure 3a); however, ST mice displayed a more robust endocrine response to stress than GT and IT mice (*P*<0.05), suggesting that they are more reactive to stress. On the other hand, corticosterone levels returned to baseline in all groups by 120 min after the stress, indicating intactness of corticosterone negative feedback regulatory mechanisms in ST animals.

High stress reactivity is linked to compromised coping behavior in unfamiliar or hostile environments.^35^ Here, ST, GT and IT mice did not show differences in emotionality or stress-coping behavior between ST, GT and IT mice, as measured by locomotor activity, interaction with a novel object and struggling versus floating times in an FST (Figure 3b–e).

In summary, the display of high stress reactivity by ST mice is not predictive of increased emotionality, a factor thought to contribute to increased vulnerability to addictive behaviors.

### Motivational behavior is intact in ST

ST rats show alterations in mesolimbic dopaminergic transmission,^36^ reflecting altered motivational state. Here, application of food retrieval test in calorie-restricted animals to assess behavioral characteristics that would inform on motivation showed that ST, GT and IT retrieved a highly rewarding food (sweetened milk) with similar latencies (Figure 3f), consumed the reward in a similar time (Figure 3g) and made a similar number of food-tray entries (Figure 3h). These findings indicate that, although ST CRs to cues predictive of food reward are associated with higher vulnerability to compulsive behaviors like addiction (see,ref. 37), ST *per se* does not equate to increased motivational drive for food reward.

### Sign-trackers do not overconsume a highly rewarding food

**Figure 4:**
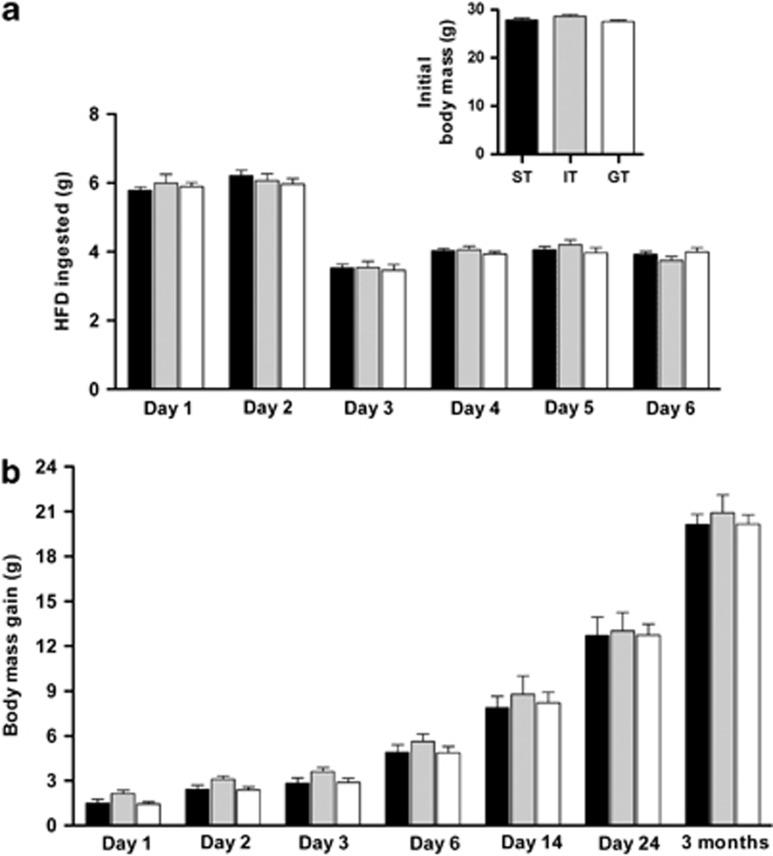
Patterns of consumption and body mass growth curves in sign-tracking (ST), goal-tracking (GT) and intermediate-tracking (IT) mice maintained on a high-fat diet (HFD). Sign-trackers (ST; *n*=13), goal-trackers (GT; *n*=11) and intermediate trackers (IT; *n*=7) were placed on a highly palatable high-fat diet, available *ad libitum*, for a period of 3 months. The consumption of the HFD was similar in all groups during the first 6 days of exposure to the HFD (**a**); note that groups did not differ in their initial body masses (inset) and showed similar gains in body mass over the 3-month duration of the experiment (**b**). The data shown are means±s.e.m.

As mentioned before, rodents prefer fat-rich diets.^31^ As ST animals are reportedly more vulnerable to compulsive behavior,^38^ we here compared the preferences of ST, GT and IT mice for an HFD versus LFD, used to control for novelty of HFD. Daily monitoring of LFD versus HFD food intake over the first 6 days of the experiment revealed that ST, GT and IT animals consumed negligible amounts of the LFD (data not shown), displaying similarly high preference for the HFD (Figure 4a). Over a 3-month exposure to HFD, all groups of mice showed body mass increases (F_6,196_=85.2; *P*⩽0.0001), albeit to the same extent (Figure 4b). Thus, ST behavior does not necessarily imply overeating of rewarding foods and proneness to overweight and/or obesity.

### Lack of morphine cross-sensitization in overweight mice

**Figure 5:**
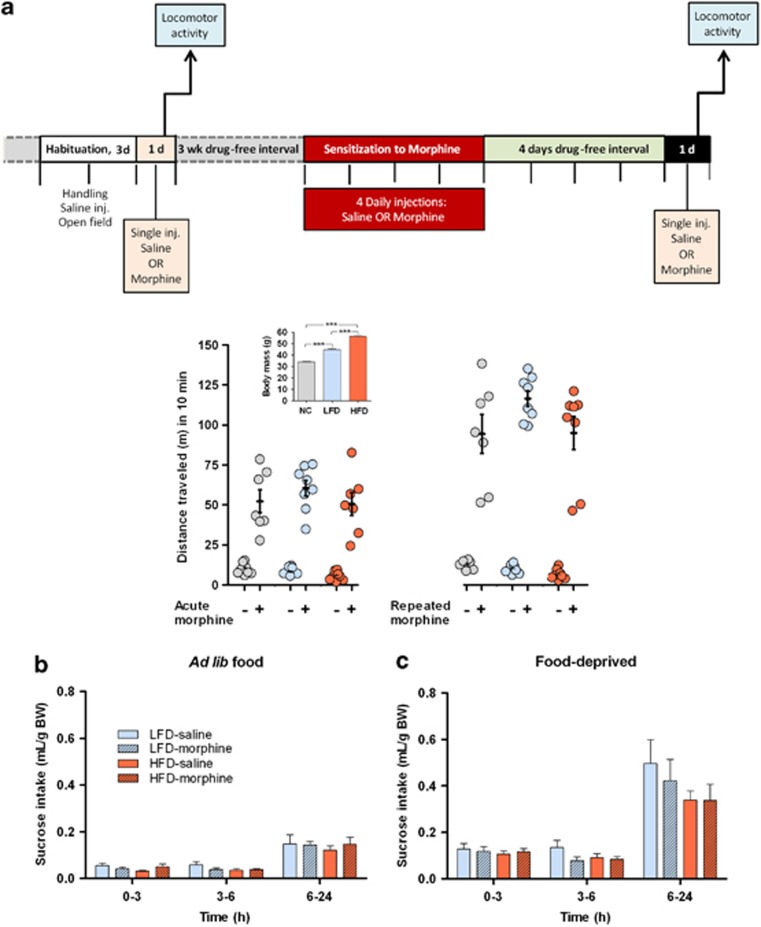
(**a**) Morphine cross-sensitization in overweight mice. The experiment was carried out in mice of varying degrees of overweight/obesity through maintenance on normal chow (NC, *n*=15), LFD (*n*=16) and HFD (*n*=16); the experimental design is shown schematically in the upper panel. Initial body masses of the different groups are shown in the inset. Animals were habituated over 3 days to the experimental procedure through handling by the experimenter and injection of saline intraperitoneally (0.2 ml); mice were then introduced into the open field (OF) test arena (5 min per day; arena specifications and test conditions are described in the legend to Figure 3). Following habituation, animals received intraperitoneal injections of either saline or morphine (20 mg kg^−1^) and returned to their home cages for 20 min before placement in the OF for 10 min during which time their locomotor activity was recorded. After a 3-week interval, mice were given four consecutive intraperitoneal injections of morphine (20 mg kg^−1^ per day) or vehicle and kept morphine-free (withdrawn) for 4 days before administration of an acute injection of morphine (20 mg kg^−1^) or saline, after which they were returned to their home cages (20 min) and then monitored for locomotor activity in the OF for over 10 min; video recordings of the latter were evaluated using ANY-maze software. Results obtained after the acute injections are shown in the left-hand panel, those after repeated morphine or saline injections are depicted in the right-hand panel. Data are shown as means±s.e.m. (**b**–**c**) Sucrose consumption test in morphine-sensitized mice in differing states of obesity. Experiments were performed in animals that were maintained on either LFD or HFD and some of which were sensitized to morphine (LFD-saline, *n*=8; LFD-morphine, *n*=8; HFD-saline, *n*=7; HFD-morphine, *n*=7). Animals were provided with solution of water and 5% sucrose; intake of sucrose was monitored at intervals over a period of 24 h, when mice were satiated (*ad libitum* food) (**b**) or food-deprived for 24 h (**c**). Depicted data are means±s.e.m. HFD, high-fat diet; LFD, low-fat diet.

Sensitization to the incentive and motivational properties of drugs of abuse is considered a primary cause of drug addiction^39^ and is accompanied by hyperresponsiveness of the mesolimbic reward pathway to dopamine, a feature found in drug addicts and overweight/obese individuals.^40^ Accordingly, we used a cross-sensitization paradigm to examine whether mice displaying varying degrees of overweight (*P*<0.0001) would display sensitization (hyperlocomotion) to a single injection of another reward-associated stimulus (morphine). Metabolic status did not influence baseline locomotor activity (10 min in OF after a saline injection) and locomotor activity was equally increased (*P*<0.0001) in all groups after a single injection of morphine (20 mg kg^−1^) (Figure 5a). After a 3-week drug-free period, the same animals received four consecutive injections of morphine (20 mg kg^−1^), followed by 4 days of withdrawal from morphine. Similar locomotor activity was displayed by all mice after a final injection of morphine (20 mg kg^−1^; Figure 5a), indicating that, irrespective of their maintenance diets (normal chow, LFD, HFD) or body mass, all animals were similarly sensitized to the opiate (*P*<0.01, compared with initial acute morphine injection).

### Morphine sensitization does not alter sensitivity to food reward

As inadequate dopaminergic transmission in the mesolimbic reward pathway appears to be a central mechanism in drug addiction,^39^ it was of interest to examine whether previous sensitization of overweight mice to an opiate alters sensitivity to a food reward (5% sucrose solution); half of the mice were previously sensitized to morphine. Morphine-sensitized and nonsensitized mice consumed similar amounts of sucrose when satiated (*P*>0.05; Figure 5b), indicating that treatment groups did not differ in terms of reward homeostasis or attribution of hedonic values to sucrose and, that sucrose liking is not a function of satiety level. Indirect evaluation of the influence of energy state on this measure was made by repeating the study in food-deprived (24 h) animals. This experiment revealed that, notwithstanding the possible stress associated with food deprivation, morphine-sensitized and nonsensitized mice ingested similar amounts of sucrose (*P*>0.05; Figure 5c).

Together, these results confirm the intactness of reward mechanisms in mice on diets that differ in reward value; moreover, they demonstrate that previous sensitization to a drug with addictive potential (morphine) does not interfere with the response to a rewarding food.

## Discussion

Eating, an essential behavior, depends on the dynamic integration of potentially conflicting peripheral and cerebral signals. Associative learning, important for acquisition of ‘liking' of foods, can evolve into conditioned, and eventually uncontrollable ‘wanting' or ‘desire' intense wanting leads to compulsive overeating in excess of physiological needs and eventually, obesity. As compulsive behavior is a characteristic of addictive behavior,^35^ a popular hypothesis is that excessive eating represents an addictive state.^19,21,23,41^

Using Pavlovian conditioning, we show that individual mice can form strong associations between a conditioned stimulus and food (cf. refs 42,43). This GT behavior contrasts with the two other robust types of CRs, namely, IT (alternation between conditioned and unconditioned stimuli) and ST (persistent responding to the conditioned stimulus). Importantly, the reward value did not influence the rate of acquisition of these CR patterns, indicating that the associative learning occurred independently of changes in motivational state. This interpretation was supported by data from independent experiments in which all mice made a similar number of food-tray entries and showed similar reward retrieval and consumption latencies. As the mice used in this work were not selected on the basis of any pre-existing behavioral or physiological traits, the behavioral responses observed cannot be attributed to the nature of the CS and US, but rather reflect natural variations in conditioned learning in general.

ST behavior is interesting in the context of our central question: can an essential function like eating transform into an addictive behavior? STs are thought to be vulnerable to compulsive behavioral disorders, including addiction, because they ‘attribute incentive salience to cues that are predictive of reward'^38^ this is exemplified by the fact that ST animals display a higher sensitivity to cocaine cues^44^ and cocaine-induced hyperlocomotion.^14^ The vulnerability of ST to addictive behavior is attributed to altered dopamine receptor expression in the ventral tegmental area-nucleus accumbens motivation-reward circuitry^36^ where dopamine receptors have an essential role in the manifestation of ST behavior.^12^ Interestingly, obese humans display reduced dopamine binding in the mesocorticolimbic reward pathway;^45,40^ this pathway is similarly activated in ‘food-addicted' (obese) and drug-addicted subjects.^11,46,47^ These associations form the backbone of the hypothesis that overeating is a type of addictive behavior.^21^

Excessive eating, especially of energy-dense foods, may be a cause or consequence of stress and the ensuing hypersecretion of glucocorticoids,^48, 49, 50^ both potentially important etiopathogenic factors in drug addiction.^51^ Although previous authors reported greater sensitivity of rats to the stressful effects of the stress induced by the autoshaping procedure itself,^38,43^ rigorous profiling in the present study did not disclose dysregulation of the dynamic regulation of the endocrine response to stress in any of the mice. Novelty-seeking, another correlate of vulnerability to drug abuse, is often associated with hyperresponsiveness, to unfamiliar environments, reflected in parallel increases in glucocorticoid secretion.^52,53^ Interestingly, the latter is associated with impaired mood and affect,^54^ conditions associated with propensity to self-administer drugs and other substances of abuse.^55^ Altogether, the above similarities between ST, IT and GT mice suggest that, unlike the situation in drug-addicted subjects,^38^ susceptibility to overeating is not directly linked to ST behavior, stress reactivity, stress-coping behavior or emotionality. It is important to note that ST behavior is highly predictive of addictive behavior has been recently challenged by observations that GT animals display two characteristics of vulnerability to addiction, namely, context-conditioned hyperactivity and context-induced reinstatement of drug-seeking behavior.^56^

Given the evidence that ST signifies risk for compulsive behavior,^37,57^ it is interesting that our ST, GT and IT mice did not display significantly different body mass gains when given a choice of HFD versus LFD over 3 months. Rather than ascribing this result to long-term homeostatic adjustments in ST animals (all groups ingested similar amounts of the HFD during the introductory phase when factors such as novelty and affect would be expected to have a significant role in shaping subsequent eating behavior), we suggest that excessive eating is triggered simply by the availability of palatable food and that ST *per se* does not predispose individuals to seek highly-rewarding foods. On the other hand, we cannot rule out (i) that HFD, which is more rewarding in terms of sensory stimulation and calorific value, induces liking, wanting and, ultimately, compulsive eating, or (ii) that metabolic and other physiological responses to the energetic and other nutritional components of HFD themselves drive eating to match physiological demands.

Like those of drugs and substances of abuse, the rewarding properties of food are mediated by dopaminergic neurons in the mesocorticolimbic pathway.^46^ The compulsive consumption of all these rewards appears to result from a hijacking of the homeostatic mechanisms that control motivational status, affect, decision-making and behavioral inhibition.^58^ However, although the subjective reward salience of addictive drugs have an important role during initiation of the addictive process, the reward salience of food is determined not only by its sensory properties but also by the subject's physiological and metabolic status. A tenable alternative to the idea that ‘food addiction' is responsible for overeating and obesity is expounded in the emerging holistic ‘hedonic theory of eating',^59,60^ which factors in the important contribution of sensory and peripheral (for example, energy balance) elements into the complex equation that determines eating and other appetitive behaviors. Specifically, hedonic theory posits that excessive consumption of a particular food results from delivery of specific sensory ‘pleasure(s)' that override homeostatic ‘stop eating' signals.

Our initial hypothesis that ST behavior can be used to identify animals that are susceptible to overeating proved false. To further examine the question of whether overeating is an addictive behavior, we borrowed the paradigm of behavioral (psychomotor) sensitization from the drug addiction field which considers such sensitization critical for the attribution of incentive salience to reward-associated stimuli.^37,39,61^ Specifically, we used a drug-food cross-sensitization paradigm (cf. refs 62, 63, 64) to discriminate between excessive intake of pleasure-giving foods and addictive drugs. Our experiments demonstrated that mice of differing body mass (normal, overweight, obese) do not display food-morphine cross-sensitization. As drug addiction may be considered to be an attempt to compensate for an underlying malfunction in reward pathways,^61,65,66^ we conducted a converse experiment to test whether morphine-sensitized mice would overconsume (substitute) a highly rewarding food (5% sucrose). Those experiments showed that morphine-sensitized and nonsensitized mice ingest similar amounts of sucrose under conditions of satiety and starvation and, further, that body mass does not influence reward consumption. Thus, (i) unlike drugs of abuse, food does not induce behavioral sensitization, and (ii) sensitization to a drug of abuse does not alter consumption of food in overweight mice. It thus appears that although the rewarding and motivational effects of food and drugs of abuse are mediated by similar neurochemical mechanisms, obesity and drug addiction represent a summation of other dysfunctional input and output pathways that lead to the emergence of two distinct disorders.

It is prudent to note that, our cross-sensitization studies were done with the opiate morphine, a prototypic drug of abuse; this contrasts with the common use of cocaine (popularly used in drug abuse research) to investigate the question of whether feeding can become addictive. Like cocaine, morphine induces behavioral sensitization, cross-sensitization to other abused drugs and conditioned place preference and, is self-administered by experimental animals.^62, 63, 64^ Monoaminergic systems are activated by morphine and cocaine, both of whose effects ultimately converge to increase dopaminergic signaling in the nucleus accumbens, albeit through different mechanisms: morphine disinhibits dopaminergic neurons in the ventral tegmental area by inhibiting γ-aminobutyric acid interneuron activity, whereas cocaine increases dopamine at nucleus accumbens terminals by inhibiting monoamine uptake.^67^ In light of these profiles, there is no *a priori* reason to expect that food-morphine and food-cocaine cross-sensitization should produce qualitatively different sensitization of the dopaminergic system, among others. Moreover, the suitability of using morphine in food–drug of abuse cross-sensitization studies has been demonstrated previously.^62, 63, 64^

Observations that similar corticolimbic pathways are activated in obese subjects and subjects who are addicted to drugs of abuse^21^ have propagated the idea that addiction to energy-dense/sweet foods underlies human obesity. However, there is growing consensus that this parallel is misleading, aptly embodied in the statement that ‘food addiction is neither enough nor necessary to develop obesity in humans'.^24^ Results from studies in animals have also contributed to the ‘addiction hypothesis of obesity'. However, critical appraisal of one of the key studies suggesting that rats can become addicted to sugar^19,25^ has been challenged on the grounds that the animals in those studies did not gain body mass, most probably because they consumed less of their standard diet, the availability of which was restricted.^20,22,26^ Another study reported compulsive eating and weight gain in rats on a cafeteria diet in which animals could choose between standard chow and a high-fat/high-sugar diet,^23^ but its conclusions do not distinguish between compulsive binge eating and overeating on one hand, and on the other, overeating which leads to obesity.^20^ Different physiological and neurobiological mechanisms are likely to underlie the two disturbed patterns of eating and notably, only binge eating shares (some) characteristics with addictive processes in humans.^68^

In summary, we demonstrate that the display of ST behavior in Pavlovian conditioning does not indicate susceptibility to overeat. This, together with our observation that cross-sensitization between food and drugs of abuse does not occur, adds important new evidence to the debate about whether eating can become an addictive behavior and lead to obesity.^22,68^ On the basis of this, we suggest that pharmacological treatments designed for drug abuse are unlikely to be effective at reducing overeating and thus, overweight and obesity. Our results may help change patients' and society's perception of overeating as a distinct disorder that does not carry the stigma still attached to addictive disorders. Nevertheless one caveat with respect to our work is that results from experiments in laboratory animals cannot be directly extrapolated to understanding human obesity: although the former eat what is provided to survive, they do not experience the natural hazards faced by free-foragers and lack the abundance and choice of foods enjoyed by humans living in industrialized societies.

## References

[bib1] Kivimäki M Lawlor DA Singh-Manoux A Batty GD Ferrie JE Shipley MJ Common mental disorder and obesity: insight from four repeat measures over 19 years: prospective Whitehall II cohort study BMJ 2009 339 b3765 19808765 10.1136/bmj.b3765 PMC2758338

[bib2] Scott KM McGee MA Wells JE Oakley Browne MA Obesity and mental disorders in the adult general population J Psychosom Res 2008 64 97 105 18158005 10.1016/j.jpsychores.2007.09.006

[bib3] Allison DB Newcomer JW Dunn AL Blumenthal JA Fabricatore AN Daumit GL Obesity among those with mental disorders: a National Institute of Mental Health meeting report Am J Prev Med 2009 36 341 350 19285199 10.1016/j.amepre.2008.11.020

[bib4] Cohen DA Neurophysiological pathways to obesity: below awareness and beyond individual control Diabetes 2008 57 1768 1773 18586908 10.2337/db08-0163 PMC2453637

[bib5] Martin-Soelch C Linthicum J Ernst M Appetitive conditioning: neural bases and implications for psychopathology Neurosci Biobehav Rev 2007 31 426 440 17210179 10.1016/j.neubiorev.2006.11.002 PMC2693132

[bib6] Saunders BT Robinson TE Individual variation in resisting temptation: implications for addiction Neurosci Biobehav Rev 2013 37 1955 1975 23438893 10.1016/j.neubiorev.2013.02.008 PMC3732519

[bib7] Weingarten HP Conditioned cues elicit feeding in sated rats: a role for learning in meal initiation Science 1983 220 431 433 6836286 10.1126/science.6836286

[bib8] Petrovich GD Ross CA Gallagher M Holland PC Learned contextual cue potentiates eating in rats Physiol Behav 2007 90 362 367 17078980 10.1016/j.physbeh.2006.09.031 PMC1892280

[bib9] Jansen A Theunissen N Slechten K Nederkoorn C Boon B Mulkens S Overweight children overeat after exposure to food cues Eat Behav 2003 4 197 209 15000982 10.1016/S1471-0153(03)00011-4

[bib10] Halford JC Boyland EJ Cooper GD Dovey TM Smith CJ Williams N Children's food preferences: Effects of weight status, food type, branding and television food advertisements (commercials) Int J Pediatric Obes 2008 3 31 38 10.1080/17477160701645152 17963122

[bib11] Rothemund Y Preuschhof C Bohner G Bauknecht H Klingebiel R Flor H Differential activation of the dorsal striatum by high-calorie visual food stimuli in obese individuals NeuroImage 2007 37 410 421 17566768 10.1016/j.neuroimage.2007.05.008

[bib12] Saunders BT Robinson TE The role of dopamine in the accumbens core in the expression of Pavlovian-conditioned responses Eur J Neurosci 2012 36 2521 2532 22780554 10.1111/j.1460-9568.2012.08217.x PMC3424374

[bib13] Saunders BT Robinson TE A cocaine cue acts as an incentive stimulus in some but not others: implications for addiction Biol Psychiatry 2010 67 730 736 20045508 10.1016/j.biopsych.2009.11.015 PMC2849872

[bib14] Flagel SB Watson SJ Akil H Robinson TE Individual differences in the attribution of incentive salience to a reward-related cue: Influence on cocaine sensitization Behav Brain Res 2008 186 48 56 17719099 10.1016/j.bbr.2007.07.022 PMC2225480

[bib15] Tomie A Grimes KL Pohorecky LA Behavioral characteristics and neurobiological substrates shared by Pavlovian sign-tracking and drug abuse Brain Res Rev 2008 58 121 135 18234349 10.1016/j.brainresrev.2007.12.003 PMC2582385

[bib16] Hernandez L Hoebel BG Feeding and hypothalamic stimulation increase dopamine turnover in the accumbens Physiol Behav 1988 44 599 606 3237847 10.1016/0031-9384(88)90324-1

[bib17] Small DM Jones-Gotman M Dagher A Feeding-induced dopamine release in dorsal striatum correlates with meal pleasantness ratings in healthy human volunteers NeuroImage 2003 19 1709 1715 12948725 10.1016/s1053-8119(03)00253-2

[bib18] Di Chiara G Imperato A Drugs abused by humans preferentially increase synaptic dopamine concentrations in the mesolimbic system of freely moving rats Proc Natl Acad Sci USA 1988 85 5274 5278 2899326 10.1073/pnas.85.14.5274 PMC281732

[bib19] Avena NM Rada P Hoebel BG Evidence for sugar addiction: behavioral and neurochemical effects of intermittent, excessive sugar intake Neurosci Biobehav Rev 2008 32 20 39 17617461 10.1016/j.neubiorev.2007.04.019 PMC2235907

[bib20] Ziauddeen H Farooqi IS Fletcher PC Obesity and the brain: how convincing is the addiction model Nat Rev Neurosci 2012 13 279 286 22414944 10.1038/nrn3212

[bib21] Volkow ND Wang G Tomasi D Baler RD Obesity and addiction: neurobiological overlaps Obes Rev 2013 14 2 18 23016694 10.1111/j.1467-789X.2012.01031.x PMC4827343

[bib22] Ziauddeen H Fletcher PC Is food addiction a valid and useful concept Obes Rev 2013 14 19 28 23057499 10.1111/j.1467-789X.2012.01046.x PMC3561707

[bib23] Johnson PM Kenny PJ Dopamine D2 receptors in addiction-like reward dysfunction and compulsive eating in obese rats Nat Neurosci 2010 13 635 641 20348917 10.1038/nn.2519 PMC2947358

[bib24] Epstein DH Shaham Y Cheesecake-eating rats and the question of food addiction Nat Neurosci 2010 13 529 531 20421898 10.1038/nn0510-529 PMC3147141

[bib25] Avena NM Gearhardt AN Gold MS Wang GJ Potenza MN Tossing the baby out with the bathwater after a brief rinse? The potential downside of dismissing food addiction based on limited data Nat Rev Neurosci 2012 13 514 22714023 10.1038/nrn3212-c1

[bib26] Peters A Does sugar addiction really cause obesity Front Neuroenergetics 2011 3 11 22275898 10.3389/fnene.2011.00011 PMC3257837

[bib27] Horner AE Heath CJ Hvoslef-Eide M Kent BA Kim CH Nilsson SR The touchscreen operant platform for testing learning and memory in rats and mice Nat Protoc 2013 8 1961 1984 24051959 10.1038/nprot.2013.122 PMC3914026

[bib28] Salomon L Lanteri C Glowinski J Tassin J Behavioral sensitization to amphetamine results from an uncoupling between noradrenergic and serotonergic neurons Proc Natl Acad Sci USA 2006 103 7476 7481 16648258 10.1073/pnas.0600839103 PMC1464364

[bib29] Jarvandi S Thibault L Booth DA Rats learn to eat more to avoid hunger Q J Exp Psychol (Hove) 2009 62 663 672 19048448 10.1080/17470210802426858

[bib30] Olausson P Kiraly DD Gourley SL Taylor JR Persistent effects of prior chronic exposure to corticosterone on reward-related learning and motivation in rodents Psychopharmacology (Berl) 2013 225 569 577 22983097 10.1007/s00213-012-2844-4 PMC3546199

[bib31] Sclafani A Oral and postoral determinants of food reward Physiol Behav 2004 81 773 779 15234183 10.1016/j.physbeh.2004.04.031

[bib32] Flagel SB Robinson TE Clark JJ Clinton SM Watson SJ Seeman P An animal model of genetic vulnerability to behavioral disinhibition and responsiveness to reward-related cues: implications for addiction Neuropsychopharmacology 2010 35 388 400 19794408 10.1038/npp.2009.142 PMC2794950

[bib33] Hearst E Jenkins HM Sign tracking: the stimulus–reinforcer relation and directed action Monograph of the Psychonomic Society: Austin, TX, USA 1974

[bib34] Boakes R Performance on learning to associate a stimulus with positive reinforcement Operant-Pavlovian Interactions Erlbaum Associates: Hillsdale, NJ, USA 67 971977.

[bib35] Sousa N Almeida OFX Wotjak CT A hitchhiker's guide to behavioral analysis in laboratory rodents Genes Brain Behav 2006 5 (Suppl 2 5 24 16681797 10.1111/j.1601-183X.2006.00228.x

[bib36] Flagel SB Watson SJ Robinson TE Akil H Individual differences in the propensity to approach signals vs goals promote different adaptations in the dopamine system of rats Psychopharmacology 2007 191 599 607 16972103 10.1007/s00213-006-0535-8

[bib37] Fineberg NA Potenza MN Chamberlain SR Berlin HA Menzies L Bechara A Probing compulsive and impulsive behaviors, from animal models to endophenotypes: a narrative review Neuropsychopharmacology 2010 35 591 604 19940844 10.1038/npp.2009.185 PMC3055606

[bib38] Flagel SB Akil H Robinson TE Individual differences in the attribution of incentive salience to reward-related cues: implications for addiction Neuropharmacology 2009 56 139 148 18619474 10.1016/j.neuropharm.2008.06.027 PMC2635343

[bib39] Robinson TE Berridge KC The incentive sensitization theory of addiction: some current issues Philos Trans R Soc Lond B Biol Sci 2008 363 3137 3146 18640920 10.1098/rstb.2008.0093 PMC2607325

[bib40] Volkow ND Wang G Telang F Fowler JS Thanos PK Logan J Low dopamine striatal D2 receptors are associated with prefrontal metabolism in obese subjects: possible contributing factors NeuroImage 2008 42 1537 1543 18598772 10.1016/j.neuroimage.2008.06.002 PMC2659013

[bib41] Zilberter T Food addiction and obesity: do macronutrients matter Front Neuroenergetics 2012 4 7 22661943 10.3389/fnene.2012.00007 PMC3362736

[bib42] Meyer PJ Lovic V Saunders BT Yager LM Flagel SB Morrow JD Quantifying individual variation in the propensity to attribute incentive salience to reward cues PLoS One 2012 7 e38987 22761718 10.1371/journal.pone.0038987 PMC3382216

[bib43] Tomie A Lincks M Nadarajah SD Pohorecky LA Yu L Pairings of lever and food induce Pavlovian conditioned approach of sign-tracking and goal-tracking in C57BL/6 mice Behav Brain Res 2012 226 571 578 22026925 10.1016/j.bbr.2011.10.021 PMC3412063

[bib44] Saunders BT Robinson TE Individual variation in the motivational properties of cocaine Neuropsychopharmacology 2011 36 1668 1676 21471956 10.1038/npp.2011.48 PMC3138662

[bib45] Wang GJ Volkow ND Logan J Pappas NR Wong CT Zhu W Brain dopamine and obesity Lancet 2001 357 354 357 11210998 10.1016/s0140-6736(00)03643-6

[bib46] Volkow ND Wang G Fowler JS Telang F Overlapping neuronal circuits in addiction and obesity: evidence of systems pathology Philos Trans R Soc Lond B Biol Sci 2008 363 3191 3200 18640912 10.1098/rstb.2008.0107 PMC2607335

[bib47] Gearhardt AN Yokum S Orr PT Stice E Corbin WR Brownell KD Neural correlates of food addiction Arch Gen Psychiatry 2011 68 808 816 21464344 10.1001/archgenpsychiatry.2011.32 PMC3980851

[bib48] Born JM Lemmens SGT Rutters F Nieuwenhuizen AG Formisano E Goebel R Acute stress and food-related reward activation in the brain during food choice during eating in the absence of hunger Int J Obes (Lond) 2010 34 172 181 19844211 10.1038/ijo.2009.221

[bib49] Maniam J Morris MJ The link between stress and feeding behaviour Neuropharmacology 2012 63 97 110 22710442 10.1016/j.neuropharm.2012.04.017

[bib50] Groesz LM McCoy S Carl J Saslow L Stewart J Adler N What is eating you? Stress and the drive to eat Appetite 2012 58 717 721 22166677 10.1016/j.appet.2011.11.028 PMC3740553

[bib51] Sinha R Jastreboff AM Stress as a common risk factor for obesity and addiction Biol Psychiatry 2013 73 827 835 23541000 10.1016/j.biopsych.2013.01.032 PMC3658316

[bib52] Piazza PV Deminière JM Le Moal M Simon H Factors that predict individual vulnerability to amphetamine self-administration Science 1989 245 1511 1513 2781295 10.1126/science.2781295

[bib53] Piazza PV Deroche V Deminière JM Maccari S Le Moal M Simon H Corticosterone in the range of stress-induced levels possesses reinforcing properties: implications for sensation-seeking behaviors Proc Natl Acad Sci USA 1993 90 11738 11742 8265619 10.1073/pnas.90.24.11738 PMC48059

[bib54] Sousa N Almeida OFX Disconnection and reconnection: the morphological basis of (mal)adaptation to stress Trends Neurosci 2012 35 742 751 23000140 10.1016/j.tins.2012.08.006

[bib55] Sinha R How does stress increase risk of drug abuse and relapse Psychopharmacology (Berl) 2001 158 343 359 11797055 10.1007/s002130100917

[bib56] Robinson TE Yager LM Cogan ES Saunders BT On the motivational properties of reward cues: individual differences Neuropharmacology 2014 76 450 459 23748094 10.1016/j.neuropharm.2013.05.040 PMC3796005

[bib57] Yager LM Robinson TE A classically conditioned cocaine cue acquires greater control over motivated behavior in rats prone to attribute incentive salience to a food cue Psychopharmacology 2013 226 217 228 23093382 10.1007/s00213-012-2890-y PMC3570662

[bib58] Kringelbach ML Stein A van Hartevelt TJ The functional human neuroanatomy of food pleasure cycles Physiol Behav 2012 106 307 316 22487544 10.1016/j.physbeh.2012.03.023

[bib59] Berridge KC Kringelbach ML Affective neuroscience of pleasure: reward in humans and animals Psychopharmacology (Berl) 2008 199 457 480 18311558 10.1007/s00213-008-1099-6 PMC3004012

[bib60] Berridge KC Kringelbach ML Neuroscience of affect: brain mechanisms of pleasure and displeasure Curr Opin Neurobiol 2013 23 294 303 23375169 10.1016/j.conb.2013.01.017 PMC3644539

[bib61] Pastor R Kamens HM McKinnon CS Ford MM Phillips TJ Repeated ethanol administration modifies the temporal structure of sucrose intake patterns in mice: effects associated with behavioral sensitization Addict Biol 2010 15 324 335 20624153 10.1111/j.1369-1600.2010.00229.x PMC2904994

[bib62] Nencini P Stewart J Chronic systemic administration of amphetamine increases food intake to morphine, but not to U50-488H, microinjected into the ventral tegmental area in rats Brain Res 1990 527 254 258 2174718 10.1016/0006-8993(90)91144-6

[bib63] Bakshi VP Kelley AE Sensitization and conditioning of feeding following multiple morphine microinjections into the nucleus accumbens Brain Res 1994 648 342 346 7922551 10.1016/0006-8993(94)91139-8

[bib64] Le Merrer J Stephens DN Food-induced behavioral sensitization, its cross-sensitization to cocaine and morphine, pharmacological blockade, and effect on food intake J Neurosci 2006 26 7163 7171 16822973 10.1523/JNEUROSCI.5345-05.2006 PMC6673946

[bib65] Vanderschuren LJ Kalivas PW Alterations in dopaminergic and glutamatergic transmission in the induction and expression of behavioral sensitization: a critical review of preclinical studies Psychopharmacology (Berl) 2000 151 99 120 10972458 10.1007/s002130000493

[bib66] Vezina P Sensitization of midbrain dopamine neuron reactivity and the self-administration of psychomotor stimulant drugs Neurosci Biobehav Rev 2004 27 827 839 15019432 10.1016/j.neubiorev.2003.11.001

[bib67] Koo JW Mazei-Robison MS Chaudhury D Juarez B LaPlant Q Ferguson D BDNF is a negative modulator of morphine action Science 2012 338 124 128 23042896 10.1126/science.1222265 PMC3547365

[bib68] de Jong JW Vanderschuren LJ Adan RA Towards an animal model of food addiction Obes Facts 2012 5 180 195 22647301 10.1159/000338292

