# Supplementary figures and images for: Pavlovian conditioning and cross-sensitization studies raise challenges to the hypothesis that overeating is an addictive behavior
Source: Transl Psychiatry. 2014 Apr 29;4(4):e387–. doi: 10.1038/tp.2014.28 (PMC4012290; doi:10.1038/tp.2014.28)

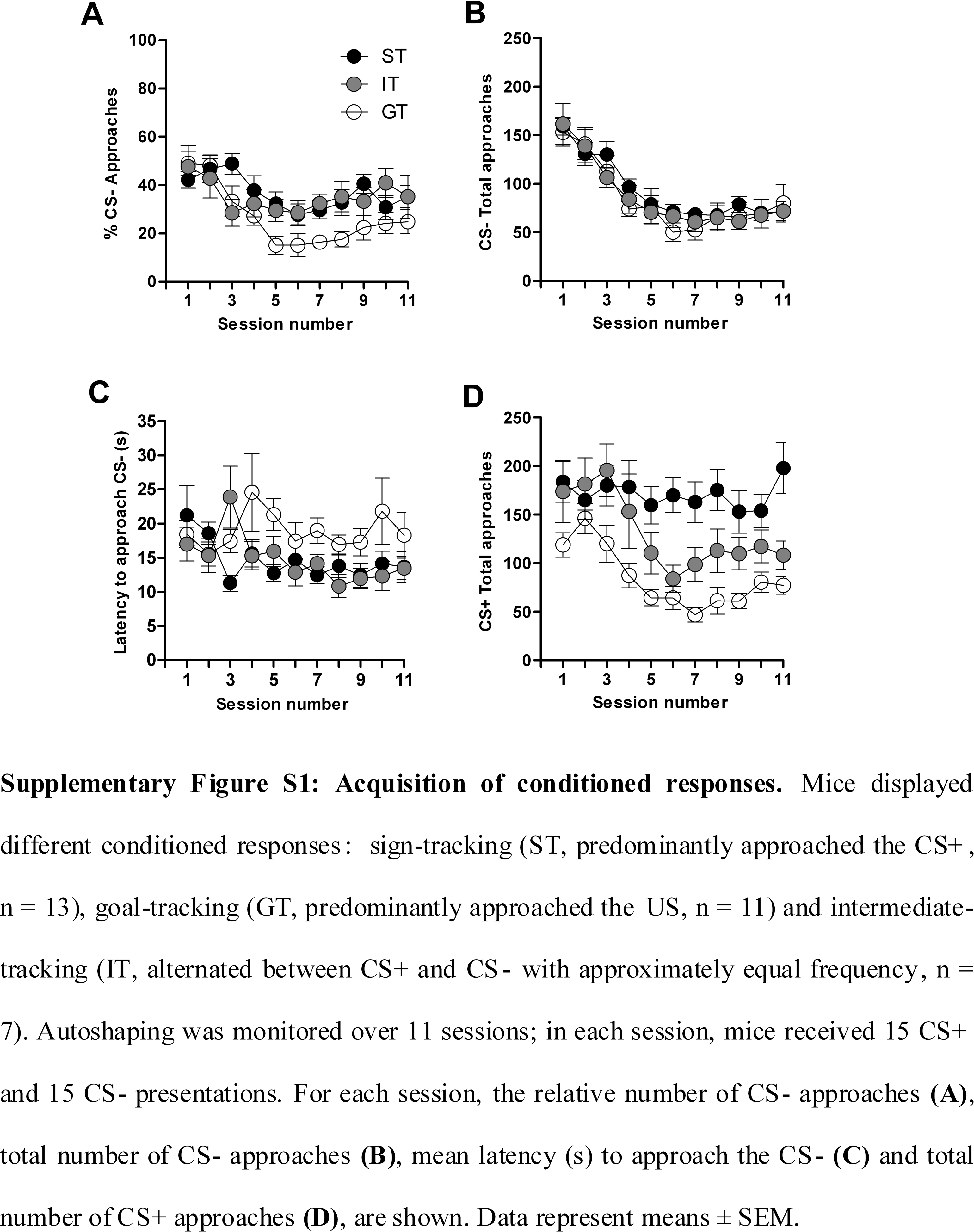

Supplement: Supplementary Figure S1 [file tp201428x1.tif]
